# Biodiversity and Multifunctional Features of Lactic Acid Bacteria Isolated From Table Olive Biofilms

**DOI:** 10.3389/fmicb.2019.00836

**Published:** 2019-04-17

**Authors:** Antonio Benítez-Cabello, Beatriz Calero-Delgado, Francisco Rodríguez-Gómez, Antonio Garrido-Fernández, Rufino Jiménez-Díaz, Francisco Noé Arroyo-López

**Affiliations:** Department of Food Biotechnology, Instituto de la Grasa, Agencia Estatal Consejo Superior de Investigaciones Científicas, Pablo de Olavide University, Seville, Spain

**Keywords:** Spanish-style green olives, natural olives, genotyping, multifunctional starters, biofilms

## Abstract

In the present study, a total of 554 lactic acid bacteria (LAB) isolates were obtained from the olive surface of Manzanilla, Gordal, and Aloreña cultivars processed as green Spanish-style or directly brined (natural) olives. The isolates obtained from industrial processes were genotyped by rep-PCR with primer GTG_5_, collecting a total of 79 different genotypes. The α-biodiversity indexes showed that the LAB diversity was higher in the biofilms on the fruits which followed the Spanish-style process than in those just brined. Sixteen genotypes had a frequency higher >1% and were identified, by multiplex PCR *recA* gene and 16S gene sequencing, as belonging to *Lactobacillus pentosus* (*n* = 13) and *Lactobacillus plantarum* (*n* = 3) species. A multivariate analysis based on a dataset with 89,744 cells, including technological (resistance to salt and pH, production of lactic acid, auto and co-aggregation with yeast species, β-glucosidase and esterase activities), and potential probiotic characteristics (survival to gastric and pancreatic digestions, resistance to antibiotics, inhibition of pathogens, presence of *bsh* genes, cholesterol removal, hemolytic, α-glucosidase, β-galactosidase, and phytase activities) showed that the 16 genotypes could be grouped into 3 great phenotypes. Thus, the genotype biodiversity in table olive biofilms was limited but, at phenotype level, it was even lower since *L. pentosus* predominated clearly (80.15% isolates). *L. pentosus* Lp13 was the genotype with the most promising characteristics for its use as a multifunctional starter, with this strain being and ubiquitous microorganism present in both natural and lye-treated olive fermentations.

## Introduction

Table olives are a traditional fermented vegetable with many centuries of history in the Mediterranean basin, where this food has a great influence on the diet and culture of many countries. The world production of table olives exceeded 2.9 × 10^6^ tones in 2017/2018 season, with more than 80% of the total output being processed by the Mediterranean leading countries Spain, Egypt, Turkey, Algeria, Italy, Greece, and Portugal (IOC, 2019). However, South America, Australia, and the Middle East are also emerging as promising producers.

The olive fruit is a fleshy drupe. It has a bitter compound (oleuropein), so olives cannot be consumed directly from the tree and need to be processed to make them palatable. Thereby, the most recognized table olive industrial processing methods are, in order of importance: (i) Spanish-style (alkali treated green olives), (ii) Californian-style (ripe olives by alkaline oxidation), and (iii) natural or directly brined olives (green, turning color or naturally black olives) (Garrido-Fernández et al., 1997).

The processing and preservation of table olives by fermentation is carried out by a combination of sugar consumption, natural acidification and salting influenced by microorganisms, which determine the flavor, safety, and quality of the final products (Arroyo-López et al., 2015). Regardless of the process, lactic acid bacteria (LAB) species play an essential role by transforming the sugars present in olive flesh into lactic which leads to rapid acidification of brines. Also, the eventual release of bacteriocins may help (Hurtado et al., 2012). *Lactobacillus plantarum* and *Lactobacillus pentosus* are the predominant species in most olive fermentations but, depending on the olive cultivar, the processing method and the geographical origin, other lactobacilli or genera can predominate or even be the most abundant species (Hurtado et al., 2012; Heperkan, 2013).

In many industries, table olive fermentations still occur spontaneously. Thus, the process is not entirely predictable and can lead to alteration and food waste (Lanza, 2013). This way, the selection of autochthonous LAB with technological and/or potential probiotic characteristics has been carried out during the last years with the objective of developing starters with application in table olives. Many of these selected microorganisms have been validated at pilot and industrial scale with promising results such us high frequencies of imposition, acidification rates, production of aromas, formation of biofilms, etc. (De Bellis et al., 2010; Ruiz-Barba and Jiménez-Díaz, 2012; Blana et al., 2014; Martorana et al., 2017; Rodríguez-Gómez et al., 2017; Chranioti et al., 2018; Pino et al., 2018). In most of the cases, the isolation of LAB was carried out exclusively from brines (Argyri et al., 2013; Bautista-Gallego et al., 2013; Botta et al., 2014; Peres et al., 2014), whilst the presence of LAB species forming biofilms on olive epidermis has been proved recently (Arroyo-López et al., 2012; Domínguez-Manzano et al., 2012; Benítez-Cabello et al., 2015). Only microorganisms with the ability to adhere to fruits epidermis could be transported to consumers during consumption, turning olives into a probiotic food if they have demonstrated functional characteristics. Besides, the application of molecular methods has shown that the biodiversity of LAB in olive brines is sensibly higher (Abriouel et al., 2011, 2012; Lucena-Padrós et al., 2014a,b; Tofalo et al., 2014; Comunian et al., 2017) than previously estimated. However, scarce studies have been carried out to determine LAB biodiversity exclusively in olive biofilms and testing their biotechnological potential (Arroyo-López et al., 2012; Domínguez-Manzano et al., 2012; Benítez-Cabello et al., 2015).

This study aimed to search, among LAB biodiversity present exclusively in olive biofilms, for isolates with multifunctional properties of interest for the fabrication of starter cultures for table olives. For this purpose, a multidisciplinary approach using molecular, biochemical, and statistical techniques was used for the selection of suitable strains, which comprises the detachment and isolation of LAB from fruit epidermis, genotyping, clustering, identification, study of their technological and probiotic features, and finally, selection of the most promising strains by multivariate analysis.

## Materials and Methods

### Olive Samples

The fruit used in the study were taken along the fermentation process (3–90 days) from 14 fermentation vessels (6 for Gordal and 8 for Manzanilla) processed as green Spanish-style, and from 10 fermentation vessels (2 for Manzanilla, 2 for Gordal, and 6 for Aloreña) of directly brined (natural) olives. The visited industries (6) were from the Sevilla and Málaga provinces (Spain) and the sampling period included three consecutive seasons (2014–2017). A total of 554 isolates (245 from Gordal, 259 from Manzanilla, and 50 from Aloreña cultivars) were obtained for molecular analysis.

### Detachment and Isolation of LAB From Olive Biofilms

Biofilms-forming LAB isolates were recovered from olive surface according to the methodology described by Benítez-Cabello et al. (2015). Briefly, fruits were removed under sterile conditions from the fermentation vessels, transported to the laboratory and transferred into sterile distilled water for 30 min for removing non-adhered cells to olive surface. Then, fruits were pitted and 25 g immediately transferred into a stomacher bag containing 75 ml of a sterile saline solution (0.9% NaCl). Pulp was homogenized for 2 min at maximum speed (300 rpm) in a stomacher model Seward 400 (Seward Medical Ltd., West Sussex, United Kingdom). Suspension of the appropriate dilutions were then spread in Man, Rogosa and Sharpe (MRS) agar selective medium (Oxoid, Basingstoke, Hampshire, United Kingdom) supplemented with 0.02% sodium azide (Sigma, St. Louis, MO, United States). After 48 h incubation at 37°C, colonies were isolated, grown again in MRS broth at 37°C for 48 h and stored at −80°C in 20% glycerol (v/v) until further analysis.

### Molecular Genotyping and Identification of LAB Isolates

DNA of the 554 LAB isolates was extracted from 1 mL of early culture in MRS broth (OD_600nm_ = 1.0) with the rapid chloroform:isoamyl alcohol method described by Ruiz-Barba et al. (2005), and further amplified by rep-PCR analysis using the GTG_5_ primer and protocol described by Gevers et al. (2001). PCR products were electrophoresed in a 2% agarose gel and finally stained with ethidium bromide (20 min). The gel was visualized under ultraviolet light using a gel analyser model Enduro^TM^ GDS (Labnet International, Inc., United States).

The resulting fingerprints were digitally captured and analyzed with the BioNumerics 6.6 software package (Applied Maths, Kortrijk, Belgium). Only bands representing amplicons between 100 and 3,000 bp in size were included in the analysis. The similarity among digitalised profiles was calculated using the Pearson correlation coefficient, and the dendrogram was generated using the Unweighted Pair Group Method using the Arithmetic Average clustering algorithm, setting a value of 0.5% optimisation and 1.25% curve smoothing. Similarity coefficient 85.0% was considered as a cut-off value to discriminate between genotypes. This cut-off value was selected by using *L. pentosus* TOMC *LAB2*, which was included in all PCR reactions as an internal control. A representative isolate from each cluster was automatically selected by a script of the BioNumerics software whose algorithm determines the fingerprint profile that share a greater similarity with the maximum number of isolates present in the cluster.

Molecular identification of predominant genotypes (>1% isolation frequency) was performed by sequencing the 16S rDNA gene using the oligonucleotide pairs 27F/1492R (Barrangou et al., 2002). The percentage of identity of the sequences was determined through a Blast analysis with the available sequences from the NCBI GenBank database^1^. Since 16S rDNA sequence analysis could not differentiate at species level within *L. plantarum* group, a multiplex PCR of the *recA* gene was carried out to discriminate between *L. pentosus*, *L. plantarum*, and *L. paraplantarum* species (Torriani et al., 2001).

### Estimation of the Biodiversity Indexes

Simpson’s index of diversity represents the probability that two individuals randomly selected from a sample will belong to different species. The value of this index ranges between 0 and 1, with values increasing as greater is the sample diversity; it is based on the formula: 1−[Σ(*n*/*N*)^2^], where “*n*” is the total number of organisms of a particular species, and “*N*” the total number of organisms of all species. The Shannon–Wiener Index is defined as *H*′ = −Σ[(*pi*) × ln (*pi*)], where *pi* is the proportion of individuals found in species *i*. The proportion (*pi*) is estimated as *pi* = *ni*/*N*, where *ni* is the number of individuals in species *i* and *N* is the total number of individuals in the community. Typical values are generally between 1.5 and 3.5, with increasing values indicating greater sample diversity (Magurran, 2004). Shannon (*H*′) and Simpson’s (1−*D*) indexes of α-diversity were calculated at the genotype level using the Scripts available in the BioNumerics 6.6 software package.

### Assessment of the Technological Potential

All technological assays described below were executed in triplicate. To study the effect of NaCl on the predominant LAB genotypes, the MRS broth was supplemented with NaCl to obtain the following final concentrations of salt in the media: 0, 5, 10, 20, 30, 40, 60, 80, 100, 120, and 160 g/L. Then, LAB growth was monitored in a Bioscreen C automated spectrophotometer (Labsystems, Helsinki, Finland) for 7 days at 30°C with a wideband filter (420–580 nm). A total of 528 growth curves were modeled for the estimation of the NIC and MIC parameters using the reparametrized Gompertz function for decay (Bonatsou et al., 2015).

To study the effect of pH on the predominant LAB genotypes, the MRS broth was modified with HCl (0.5 N) to obtain the following pH in the medium (2, 3, 4, 5, 6, 7, 8, 9, 10, 11, and 12). Then, as in the previous case, LAB growth was monitored in a Bioscreen C automated spectrophotometer (Labsystems, Helsinki, Finland) for 7 days at 30°C with a wideband filter (420–580 nm). A total of 528 growth curves were modeled for the estimation of pH cardinal parameters (pH_opt_, pH_max_, and pH_min_) using the cardinal model with inflection proposed by Oscar (2002).

To evaluate the production of lactic acid by the predominant LAB genotypes, isolates were grown in 25 mL of MRS broth supplemented with 6% NaCl (a similar concentration obtained in olive brines). After 48 h incubation at 37°C, suitable dilutions were made and plated on MRS agar to check the population level, reaching all strains similar final concentrations (∼8 log_10_ CFU/mL). The percentage of titratable acidity was measured by using a Titroprocessor model 670 (Metrohm, Switzerland). The titratable acidity was expressed in g of lactic acid/100 mL.

Co-aggregation ability of the LAB genotypes with yeasts (*Debaromyces etchellsii* Y24 and *Candida boidinii* Y5) was studied following the protocol proposed by Toledo-Arana et al. (2001), which include co-incubation of the LAB and yeasts cultures, washing, staining with crystal violet and solubilization with ethanol-acetone. The OD_595_ was determined using a spectrophotometer SPECTROstar^^®^^ Nano (BMG Labtech). These strains were chosen because of their weak (Y24) and high (Y5) ability to form biofilms with table olive *Lactobacillus* strains (León-Romero et al., 2006).

The ability to produce enzymes of technological interest (esterase and β-glucosidase activities) by the LAB strains was also studied. These activities were evaluated by measuring the amount of *p*-nitrophenol liberated from different chromogenic substrates (*4*-nitrophenyl butyrate and *4*-nitrophenyl-β-D-glucoside) according to protocols described by Manzanares et al. (1998); Rodríguez-Gómez et al. (2012), Phan et al. (2013), and Bonatsou et al. (2015). The concentration of liberated *p*-nitrophenol was estimated from the absorbance obtained at 420 nm in a spectrophotometer (Cary1E UV-vis, Varian INC., Palo Alto, CA, United States) using the corresponding blank for each case. Results were expressed as the amount of enzyme liberating 1 nmol of *p*-nitrophenol per hour and milliliter (nmol ⋅ h^−1^ ⋅ mL^−1^) under the assay conditions for the cellular fraction.

### Assessment of the Probiotic Potential

All probiotic assays described below were executed in triplicate. In all cases, results were compared with the well-known species *Lactobacillus casei* var. Shirota and *Lactobacillus rhamnosus* GG used as the control. The resistance of the LAB strains to simulated sequential *in vitro* gastric (2.5 h) and pancreatic (3 h) digestions were studied following the protocol described by Bautista-Gallego et al. (2013) at 37°C in an orbital shaker (150 rpm) to simulate the peristaltic movements.

The ability of predominant LAB genotypes to reduce cholesterol in the medium was evaluated following the protocol described by Kourelis et al. (2010), but with slight modifications. Cholesterol concentrations in the medium were measured using a commercial kit (BioSystems, Barcelona, Spain). Briefly, 1 ml on an overnight LAB culture was centrifuged at 9,000 × *g* for 10 min and washed twice with 0.9% of a saline buffer. The pellet was then re-suspended in the same buffer and incubated during 2 h at room temperature (starvation phase). After that, 20 μl of the suspension was inoculated in 230 μl of MRS broth supplemented with 3 g/L of Oxgall (Fluka Analytical, St. Louis, MO, United States) and 0.225 g/L of cholesterol, and was incubated at 37°C in an orbital shaker at 200 rpm for 48 h. Then, the samples were centrifuged again, and the pellet was discarded. Finally, cholesterol was measured according to manufacturer instructions at 500 nm using a UV spectrophotometer (Agilent Technologies, Santa Clara, CA, United States). MRS broth + Oxgall without cholesterol were used for every sample as a control. A standard curve representing absorbance versus diverse cholesterol concentrations was obtained.

Quantitative bile salt hydrolase activity from the LAB strains was first detected following the protocol of Zago et al. (2011) with slight modifications. Briefly, overnight cultures of the bacteria were spotted on MRS agar plates containing 0.37 g/L CaCl_2_ and 0.5% of the sodium salt of glycodeoxycholic acid (GDCA) (Sigma-Aldrich). Plates were incubated at 37°C for 72 h. Then, the presence of *bsh1* and *bsh2* genes was tested after DNA extraction. *Bsh1* gen was amplified using the primers LpBsh1F/R (5′-GGATTACTAGACATGTGTACTGCC-3′/5′-GCCAGCCATTGGAACTTACTCTG-3′) (Lambert et al., 2008). *Bsh2* gen was amplified using the primers Bsh2F/R (5′-ATGTGTACCAGCCTAACTTATACCAATAGCCACGG-3′/5′-TTAGCGTGCCGTGGGTAGTGTCGCGACATCTGCGG-3′) which were designed according to the genes sequences available in NCBI GenBank for *L. pentosus* strains described by Calero-Delgado et al. (2018). PCR amplifications were carried out using for *bsh1* gene an initial denaturation step at 94°C for 4 min, followed by 35 cycles each consisting of a denaturation step at 95°C for 15 s, an annealing step at 48°C for 30 s, and an extension step at 72°C for 45 s, with a final extension step at 72°C for 6 min. In the case of *bsh2* gene, the PCR conditions were initial denaturation step at 95°C for 4 min, followed by 35 cycles each consisting of a denaturation step at 95°C for 15 s, an annealing step at 69°C for 30 s, and extension step at 72°C for 35 s, with a final extension step at 72°C for 6 min.

Hemolytic test for predominant LAB genotypes was carried out following the protocol described by Da Silva Ferrari et al. (2016). Briefly, an aliquot of an overnight culture of each LAB genotype was plated onto the Agar base (Oxoid) supplemented with 5% of defibrinated whole horse blood (Sigma) using the loop exhaustion technique. After incubation at 37°C for 24 h, the hemolytic activity of the strains was determined by observing a clear zone around the colony, which indicated a complete inhibition of the medium (β-hemolysis), a green zone or darkening of the medium, which indicated a partial hemolysis (α-hemolysis) or no inhibition zone (γ-hemolysis). *Enterococcus faecium LGM 16170* from the BCCM*/*LMG Bacteria collection was used as positive control.

The pathogen inhibition capacity of the LAB strains was evaluated using the agar well diffusion test described by Bautista-Gallego et al. (2013) with slight modifications. A lawn of BHI or Nutritive soft agar (10 g/L) medium containing 10^5^ CFU/mL of *Listeria monocytogenes NTC10357* or *Escherichia coli NTC43894* were poured onto Petri dishes. After solidification, a hole was made in the center of the plate. 100 μl of a 48 h of cell-free MRS broth of the different LAB strains was inoculated and allowed to diffuse at 4°C for 30 min. To verify the nature of the possible inhibitory effect, aliquots of untreated supernatant, treated with 10–25 mg/ml proteinase K (Sigma) and supernatant neutralized with 0.1 M KOH were analyzed. Plates were examined for halos around the hole after incubation at 37°C for 24 h.

The antibiotic susceptibility of the LAB isolates was assessed through the disk diffusion method. Briefly, 100 μl of 8 log_10_ CFU/mL LAB culture was inoculated in 4 ml of MRS 0.75% agar and spread in plates. Once dried, antibiotics disk (Liofilchem, Italy) were applied to the surface of the plates, including erythromycin (15 μg), tetracycline (30 μg), gentamicin (10 μg), penicillin (10 μg), nalidixic acid (30 μg), ampicillin (10 μg), streptomycin (10 μg), vancomycin (30 μg), chloramphenicol (30 μg), clindamycin (2 μg), kanamycin (30 μg), and cefotaxime (30 μg). Inhibition-zone diameters were measured after 24 h incubation at 37°C and compared with known standard given by the Clinical and Laboratory Standard Institute (CLSI) for antimicrobial susceptibility testing (Clinical and Laboratory Standards Institute, 2012) as described by Charteris et al. (2000). Results were expressed in terms of resistance (R), intermediate (I) and susceptible (S) according to the diameter halo obtained.

The ability to produce enzymes of probiotic interests by the predominant LAB genotypes was also studied, specifically their capacity to produce the α-glucosidase, β-galactosidase, and phytase enzymes. The activities were evaluated by measuring the amount of *p*-nitrophenol liberated from different chromogenic substrates (*4*-nitrophenyl-α-D-glucoside, *4*-nitrophenyl-β-D-galactosidase), while phytase activity was evaluated by measuring the release of inorganic phosphorus from sodium phytate (Haros et al., 2005; Bonatsou et al., 2015). The concentration of liberated *p*-nitrophenol was estimated from the absorbance obtained at 420 nm in a spectrophotometer (Cary1E UV-vis) using a suitable blank for each case, while inorganic phosphorus was measured at 405 nm. One unit of enzymatic activity was defined as the amount of enzyme liberating 1 nmol of inorganic phosphorous or *p*-nitrophenol per hour and milliliter (nmol ⋅ h^−1^ ⋅ mL^−1^) under the assay conditions for the cellular fraction.

### Statistical Analysis

Significant differences among LAB strains for the different technological and probiotic tests assayed were determined by an analysis of variance using the one-way ANOVA module of Statistica 7.1 software (Statsoft Inc., Tulsa, OK, United States) and the Scheffé *post hoc* comparison test. A multivariate analysis was also performed for detecting overall similarity between predominant genotypes and characteristics (technological and probiotic features). The study comprised a cluster analysis based on the Euclidean distance, using the Ward method, and a bicluster which grouped simultaneously according to characteristics and genotypes. For multivariate analysis, the plugin XLSTAT (v. 2017) and the MultiBiplot package (Vicente Villardón, 2016) were used.

## Results

### LAB Biodiversity in Olive Biofilms

**FIGURE 1:**
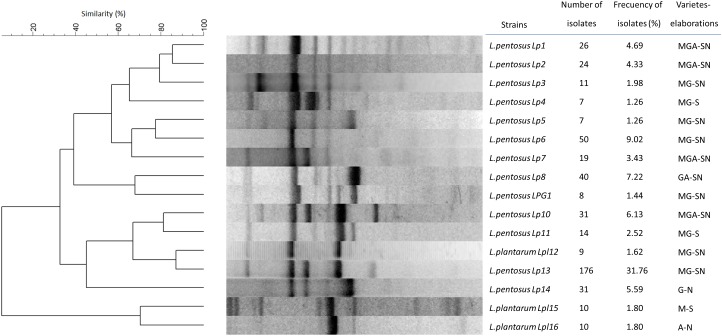
Dendrogram obtained for the rep-PCR profiles obtained with GTG_5_ primer for the predominant (>1% frequency) LAB genotypes randomly isolated from biofilms of different table olive processing. The dendrogram was built after selecting a representative genotype from each cluster by bioinformatics analysis with the Bionumerics 6.6 software package. M, G, and A stand for the type of olive cultivar (Manzanilla, Gordal, and Aloreña, respectively), while S and N refer to the type of elaboration (Spanish-Style or natural).

In this work, a total of 554 LAB isolates were obtained exclusively from olive biofilms of different industries, cultivars and processing methods. LAB isolates were first genotyped by rep-PCR analysis with GTG_5_ primer. After clustering analysis, a total of 79 different genotypes were obtained for a cut-off value of 85%, but only 16 genotypes had a frequency higher >1%, which accounted for the 85.39% of the total population (*n* = 473). Figure 1 shows the dendrogram built exclusively for these 16 predominant genotypes, using a representative isolate selected by the bioinformatics software for each genotype. The dominant genotype was Lp13 (31.77%), with this strain being a ubiquitous genotype found in practically all types of elaborations (except in Aloreña green natural olives), followed by genotype Lp6 (9.03%). The rest of the predominant genotypes had an isolation frequency ranging from 1.26 (Lp4 and Lp5) to 7.22% (Lp8). Lp1, Lp2, Lp7, and Lp10 were the most disseminated genotypes being present in all type of elaborations and varieties (Figure 1). The predominant 16 genotypes were then identified by molecular methods using sequencing of 16S rDNA gene and multiplex PCR of the *recA* gene (data not shown). Thirteen of these genotypes were assigned to *L. pentosus* species (80.15%), including Lp13 and Lp6, while the other three were identified as *L. plantarum* (5.24%). Thus, the interspecific biodiversity in the sampled population was relatively low, because only one species (*L. pentosus*) accounted for 80.15% of the total identified isolates. Conversely, this species had the highest intra-specific diversity, with 13 of the 16 predominant genotypes. The further technological and probiotic characterisation was carried out exclusively with these 16 predominant genotypes.

**FIGURE 2:**
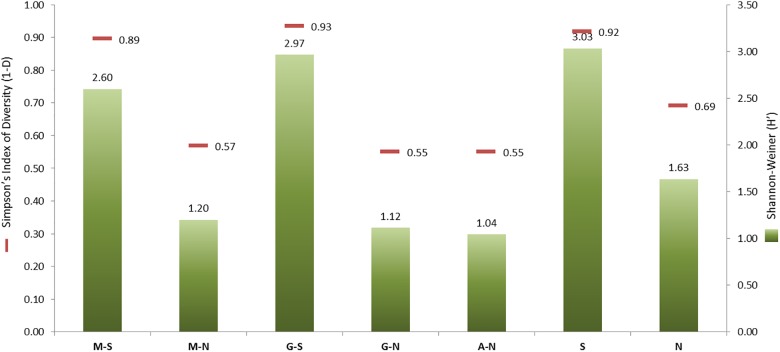
Shannon–Weiner (*H*′) and Simpson’s Indexes of Diversity (1–*D*) generated after grouping the genotypes obtained by processing style [Spanish-style (S) and natural, directly brined olives (N)] or the combinations of them with the olive cultivar [Spanish-style Manzanilla, M-S; directly brined Manzanilla, M-N; Spanish-style Gordal, G-S; directly brined Gordal, G-N; and directly brined Aloreña, A-N]. The number of isolates in every group of samples was: M-S, 155; M-N, 104; G-S, 127; G-N, 118; A-N, 50; S, 282; N, 272.

Figure 2 shows the Shannon–Weiner (*H*′) and Simpson (1−*D*) α-diversity indexes obtained for the total population (79 genotypes) as a function of processing type (Spanish-style or natural) and olive cultivar (Manzanilla, Gordal, and Aloreña) *versus the* type of processing. The total of isolates obtained for the Spanish-style (*n* = 282) and directly brined (natural) olives (*n* = 272) were very similar. However, the α-diversity indexes were higher for the Spanish-style (1−*D* = 0.92, *H*′ = 3.03) compared to the natural olives (1−*D* = 0.69, *H*′ = 1.63). These data were also confirmed when isolates were classified according to olive cultivar and processing method. Spanish-style Manzanilla and Gordal cultivars had higher biodiversity indexes than when processed as directly brined olives (Figure 2), albeit the numbers of isolates in both cases were comparable (104 and 155, respectively). The lowest α-diversity indexes were obtained for Aloreña olives processed as directly brined olives, followed by Gordal and Manzanilla natural olives.

### Technological Tests

Tables 1, 2 show the results obtained for the different technological tests assayed, according to the 16 selected LAB genotypes found. Modeling of the resistance and susceptibility of the strains to NaCl showed a good fit, with an *R*^2^ usually above 0.97 (data not shown). The range of the NIC values (susceptibility) among LAB strains ranged from 43.5 (*Lp3*) to 73.5 g/L (*Lp11*), while the MIC value (resistance) ranged from 101.2 (*Lp14*) to 131.5 g/L (*Lpl16*), with significant differences among strains according to the Scheffé *post hoc* comparison test (Table 1). Thereby, the strain with the highest overall salt resistance was *L. pentosus* Lp6. Table 1 also shows the cardinal pH parameters obtained for the 16 genotypes after fitting growth curves with the Oscar model (2002), with an *R*^2^ usually above 0.98 (data not shown). The pH_min_ ranged from 0.40 (strain Lp8) to 1.70 (Lp3), the pH_opt_ ranged from 5.96 (Lp3) to 7.96 (strains Lp8 and Lp11), while the pH_max_ ranged from 10.22 (Lp1) to 12.37 (strain Lpl16). Because the range of pH studied was from 2 to 12, values extrapolated by the model outside these limits should be considered with caution. According to these data, Lp3 was one of the most acidophilus strains (because of its low pH_max_ and pH_opt_ for growth), while Lp7 was one of the most alkaline strains (high pH_max_ and pH_opt_ for growth). Not significant differences among genotypes were found for the production in laboratory medium of lactic acid, with values ranging from 1.66 (Lp3) to 1.86% (strain Lp13) (Table 1).

**Table 1:** Technological characteristics in laboratory medium for the 16 predominant LAB genotypes obtained in the present study.

	NaCl resistance		pH cardinal values			
Strains	NIC (g/l)	MIC (g/l)	pH_max_	pH_min_	pH_opt_	Free acidity (%)
*Lp1*	64.08 (2.42)^a,b^	107.54 (3.33)^a,b,c^	10.22 (0.01)^a^	1.32 (0.02)^c,d^	7.07 (0.04)^e,f,g^	1.69 (0.08)^a^
*Lp2*	61.19 (3.61)^a,b^	105.60 (0.99)^a,b^	11.24 (0.05)^b,c^	1.49 (0.06)^c,d,e^	6.47 (0.09)^a,b,c,d^	1.76 (0.19)^a^
*Lp3*	43.51 (2.25)^a^	127.00 (4.38)^c,d^	11.07 (0.03)^b^	1.70 (0.03)^e^	5.96 (0.10)^a^	1.66 (0.09)^a^
*Lp4*	61.22 (3.72)^a,b^	105.76 (0.14)^a,b^	11.36 (0.02)^b,c,d^	1.29 (0.13)^c,d^	7.03 (0.10)^d,e,f,g^	1.78 (0.23)^a^
*Lp5*	69.52 (10.58)^b^	111.08 (6.46)^a,b,c,d^	11.37 (0.09)^b,c,d^	1.15 (0.04)^b,c^	6.86 (0.12)^c,d.e,f,g^	1.78 (0.16)^a^
*Lp6*	71.12 (4.83)^b^	130.08 (5.05)^e^	10.24 (0.03)^a^	1.51 (0.07)^c,d,e^	6.36 (0.14)^a,b,c^	1.66 (0.22)^a^
*Lp7*	64.28 (5.75)^a,b^	101.92 (3.36)^a,b^	12.36 (0.06)^e^	1.39 (0.07)^c,d,e^	7.12 (0.17)^f,g,h^	1.75 (0.32)^a^
*Lp8*	57.69 (4.18)^a,b^	102.38 (0.41)^a,b^	11.19 (0.06)^b^	0.40 (0.18)^a^	7.68 (0.16)^h^	1.78 (0.15)^a^
*LPG1*	61.10 (6.15)^a,b^	117.09 (6.17)^a,b,c,d,e^	12.03 (0.02)^c,d,e^	1.38 (0.07)^c,d,e^	6.62 (0.09)^b,c,d,e,f^	1.80 (0.14)^a^
*Lp10*	54.33 (2.71)^a,b^	125.22 (5.72)^c,d,e^	11.27 (0.03)^b,c,d^	1.43 (0.05)^c,d,e^	7.28 (0.13)^g,h^	1.79 (0.08)^a^
*Lp11*	73.47 (2.06)^b^	99.90 (3.11)^a^	11.20 (0.02)^b^	0.90 (0.10)^b^	7.68 (0.11)^h^	1.76 (0.09)^a^
*Lpl12*	67.17 (0.77)^b^	119.37 (3.55)^b,c,d,e^	11.25 (0.05)^b,c,d^	1.51 (0.04)^c,d,e^	6.84 (0.10)^c,d,e,f,g^	1.76 (0.21)^a^
*Lp13*	59.17 (4.13)	105.60 (4.40)^a,b^	12.24 (0.55)^e^	1.60 (0.05)^d,e^	6.61 (0.20)^b,c,d,e,f^	1.86 (0.15)^a^
*Lp14*	66.92 (1.31)^b^	101.18 (0.78)^a^	11.35 (0.03)^b,c,d^	1.39 (0.01)^c,d,e^	6.43 (0.03)^a,b,c^	1.75 (0.10)^a^
*Lpl15*	58.25 (9.02)^a,b^	106.99 (5.95)^a,b^	12.23 (0.01)^e^	1.52 (0.03)^d,e^	6.35 (0.09)^a,b,c^	1.80 (0.13)^a^
*Lpl16*	55.15 (1.02)^a,b^	131.54 (2.26)^e^	12.37 (0.05)^e^	1.65 (0.07^d,e^	6.64 (0.04)^b,c,d,e,f^	1.78 (0.23)^a^

*Values are expressed as mean and standard deviation (in parentheses) obtained from triplicate experiments. Different superscript letters, within the same column, means significant statistical difference (p ≤ 0.05) according to the Scheffé post hoc comparison test. NIC and MIC values for NaCl, cardinal parameters for pH, and production of titratable acidity.*

**Table 2:** Technological characteristics for the 16 predominant LAB genotypes obtained in the present study.

	Auto and co-aggregation with yeasts (OD_595 nm_)			Enzymatic activity (U)nmol^∗^ml^−1∗^h^−1^
Strains	Auto-aggregation	Co-aggregation Y5	Co-aggregation Y24	Esterase
*Lp1*	0.78 (0.34)^a,b^	1.15 (0.38)^a^	2.76 (0.76)^b,c,d^	43.89 (9.93)^a^
*Lp2*	0.51 (0.10)^a^	0.65 (0.10)^a^	0.97 (0.41)^a^	45.25 (15.31)^a^
*Lp3*	1.18 (0.43)^a,b,c^	0.57 (0.15)^a^	1.40 (0.38)^a,b^	24.01 (2.75)^a^
*Lp4*	3.50 (0.00)^e^	3.50 (0.00)^c^	3.50 (0.00)^d^	17.92 (5.28)^a^
*Lp5*	3.50 (0.00)^e^	3.50 (0.00)^c^	3.50 (0.00)^d^	40.06 (4.23)^a^
*Lp6*	0.72 (0.11)^a,b^	3.50 (0.00)^c^	0.68 (0.06)^a^	13.21 (0.51)^a^
*Lp7*	1.82 (1.04)^b,c,d^	0.92 (0.45)^a^	0.97 (0.44)^a^	7.58 (0.46)^a^
*Lp8*	3.50 (0.00)^e^	2.57 (0.36)^b^	2.76 (0.34)^b,c,d^	45.54 (7.26)^a^
*LPG1*	3.50 (0.00)^e^	3.03 (0.44)^b,c^	3.46 (0.08)^d^	182.69 (65.60)^b^
*Lp10*	0.89 (0.25)^a,b^	0.42 (0.04)^a^	0.93 (0.56)^a^	20.61 (2.83)^a^
*Lp11*	2.18 (0.83)^c,d^	1.09 (0.41)^a^	1.91 (0.86)^a,b,c^	54.08 (15.61)^a^
*Lpl12*	0.76 (0.22)^a,b^	0.51 (0.09)^a^	0.58 (0.10)^a^	18.81 (1.30)^a^
*Lp13*	1.63 (0.24)^a,b,c^	3.43 (0.14)^c^	3.10 (0.65)^c,d^	217.23 (28.84)^b^
*Lp14*	2.97 (0.51)^d,e^	3.50 (0.00)^c^	3.25 (0.39)^c,d^	36.78 (7.24)^a^
*Lpl15*	1.48 (0.28)^a,b,c^	0.93 (0.25)^a^	1.85 (0.89)^a,b,c^	17.61 (0.83)^a^
*Lpl16*	0.89 (0.21)^a,b^	0.57 (0.10)^a^	3.50 (0.00)^d^	6.96 (2.95)^a^

*Values are expressed as mean and standard deviation (in parentheses) obtained from triplicate experiments. Different superscript letters, within the same column, means significant statistical differences (p ≤ 0.05) according to Sheffe post hoc comparison test. Values for auto and co-aggregation with yeast species Candida boidinii Y5 and Debaryomyces etchellsii Y24 as well as esterase activity.*

Genotype Lp2 showed the lowest auto-aggregation value (OD_595_ = 0.51), while strains Lp4, Lp5, Lp8, and LPG1 had the highest (OD_595_ = 3.50, which is the saturation limit of the spectrophotometer). Regarding co-aggregation in the presence of eukaryotes, genotypes Lp10 and Lpl12 had low values for both yeast species (OD_595_ < 1.0), whereas strains Lp4, Lp5, LPG1, Lp13, and Lp14 showed high values (OD_595_ > 3.0, see Table 2). Except for Lp1 (10.66 nmol ⋅ h^−1^ ⋅ mL^−1^) and Lp7 (26.03 nmol ⋅ h^−1^ ⋅ mL^−1^), none of the LAB strains exhibited β-glucosidase activity but, conversely, all of them showed esterase activity, especially LPG1 (182.69 nmol ⋅ h^−1^ ⋅ mL^−1^) and Lp13 (217.23 nmol ⋅ h^−1^ ⋅ mL^−1^) genotypes (see Table 2).

### Probiotic Tests

Tables 3–5 show the results of the different probiotic tests assayed according to the 16 selected LAB genotypes. Among them, the strain Lpl15 was the isolate with the highest overall survival (excluding the probiotic microorganisms used as a control) to both gastric and pancreatic digestions (37.07 and 34.59%, respectively), while Lp10 was the most sensitive strain to the *in vitro* digestions (0.00% survival). All the strains showed the ability to reduce the cholesterol in the medium but with significant statistical differences among them. The values ranged from 13.09 (Lp5) to 38.42% (Lpl15), with levels even higher than probiotic controls LcS and LrGG. The presence of *bsh1* gene (but not *bsh2*) was detected exclusively in the *L. plantarum* strains (Lpl12, Lpl15, and Lpl16), while the *bsh2* gene was found exclusively in the *L. pentosus* strains Lp2, Lp4, Lp7, Lp10, Lp13, and Lp14.

**Table 3:** Probiotic characteristics for the 16 predominant LAB genotypes obtained in the present study.

Strains	% Survival gastric digestion	% Survival pancreatic digestion	% Cholesterol removal
*Lp1*	2.60 (0.78)^a,b^	20.29 (15.38)^a,b^	20.82 (1.90)^a,b,c^
*Lp2*	0.59 (0.04)^a,b^	73.29 (18.79)^b^	13.44 (1.90)^a^
*Lp3*	0.03 (0.01)^a,b^	0.42 (0.18)^a^	14.02 (4.06)^a^
*Lp4*	7.43 (2.45)^a,b,c^	6.85 (2.45)^a^	30.30 (2.66)
*Lp5*	11.88 (0.05)^a,b,c,d^	0.02 (0.01)^a^	13.09 (1.98)^a^
*Lp6*	10.84 (3.81)^a,b,c,d^	22.07 (21.24)^a,b^	20.29 (2.90)^a,b,c^
*Lp7*	5.41 (2.87)^a,b^	0.91 (0.81)^a^	30.29 (1.56)^b,c,d^
*Lp8*	0.00 (0.00)^a,b^	6.82 (9.64)^a^	18.31 (0.10)^a,b^
*LpG1*	2.60 (0.00)^a,b^	23.79 (3.85)^a,b^	25.31 (1.34)^a,b,c,d^
*Lp10*	0.00 (0.00)^a,b^	0.00 (0.00)^a^	27.43 (2.38)^a,b,c,d^
*Lp11*	5.31 (2.00)^a,b^	0.00 (0.00)^a^	23.91 (0.51)^a,b,c,d^
*Lpl12*	1.47 (0.74)^a,b^	12.23 (2.48)^a^	32.75 (3.38)^b,c,d^
*Lp13*	0.23 (0.01)^a,b^	7.43 (2.79)^a^	25.29 (0.98)^a,b,c,d^
*Lp14*	1.12 (0.24)^a,b^	0.90 (0.09)^a^	22.74 (4.49)^a,b,c^
*Lpl15*	37.07 (2.07)^d,e^	34.59 (2.57)^a,b^	38.42 (2.84)^d^
*Lpl16*	35.00 (5.99)^c,d,e^	13.82 (0.21)^a^	35.03 (2.33)^c,d^
*LcS*	25.34 (16.31)^b,c,d,e^	35.48 (19.64)^a,b^	32.31 (1.41)^b,c,d^
*LrGG*	56.32 (10.18)^e^	28.82 (0.36)^a,b^	32.80 (2.36)^b,c,d^

*Values are expressed as mean and standard deviation (in parentheses) obtained from duplicated experiments. Different superscript letters, within the same column, means significant statistical differences (p ≤ 0.05) according to Sheffé post hoc comparison test. Percentage of survival to simulated gastric, pancreatic digestions, and percentage of the cholesterol removal in the medium. Data were compared with the recognized probiotic microorganisms Lactobacillus casei Shirota (Lcs) and Lactobacillus rhamnosus GG (LrGG).*

β-hemolysis activity was detected for all *L. plantarum* genotypes (Lpl12, Lpl15, and Lpl16), but not for any of the 13 *L. pentosus* genotypes studied. All genotypes assayed were able to produce the inhibition of the food-borne pathogens *E. coli* and *L. monocytogenes* but, in general, the inhibition halo was broader for *E. coli* (ranged from 21.0 to 24.0 mm) than for *L. monocytogenes* (ranged from 12.00 to 16.00 mm). This inhibition was not mediated by the presence of bacteriocins (data not shown). Lpl12 was the genotype with the highest inhibition halo for both pathogens (see Table 4). A total of 9 genotypes did not exhibit α-glucosidase activity but, on the contrary, genotypes Lp1 and especially Lp11 showed high values (>200.00 nmol ⋅ h^−1^ ⋅ mL^−1^). A total of 4 genotypes did not exhibit β-galactosidase activity, but genotypes Lp8 and Lp13 showed high values (>100.00 nmol ⋅ h^−1^ ⋅ mL^−1^). On the contrary, phytase activity was widespread among the 16 genotypes assayed. This activity ranged from 1197.50 (Lp1) to 81,739.88 nmol ⋅ h^−1^ ⋅ mL^−1^ (Lp13), showing the strain LPG1 also high values (see Table 4).

**Table 4:** Probiotic characteristics for the 16 predominant LAB genotypes obtained in the present study.

	Pathogens inhibition		Enzymatic activities (U)nmol^∗^ml^−1∗^h^−1^		
Strains	*Ec*	*Lm*	β-galactosidase	Phytase	α-glucosidase
*Lp1*	21.00	12.00	0.00 (0.00)^a^	1197.50 (74.35)^a^	208.70 (24.61)^e^
*Lp2*	24.00	15.00	55.25 (18.87)^a,b,c^	11946.49 (3861.70)^a,b^	0.00 (0.00)^a^
*Lp3*	22.00	0.00	53.08 (1.31)^a,b,c^	7674.71 (938.30)^a,b^	126.38 (4.13)^c,d^
*Lp4*	23.00	15.00	47.65 (11.39)^a,b,c^	3638.42 (971.55)^a,b^	70.68 (22.15)^b,c^
*Lp5*	23.00	12.00	0.00 (0.00)^a^	18242.12 (2570.98)^a,b^	0.00 (0.00)^a^
*Lp6*	23.00	13.00	20.68 (2.91)^a^	5089.82 (155.69)^a,b^	0.00 (0.00)^a^
*Lp7*	24.00	13.00	33.05 (6.48)^a,b^	4024.19 (390.16)^a,b^	50.68 (6.35)^a,b^
*Lp8*	24.00	13.00	127.08 (31.95)^b,c^	28021.32 (4163.95)^b^	0.00 (0.00)^a^
*LPG1*	24.00	14.00	314.28 (95.59)^d^	54246.34 (17135.20)^c^	0.00 (0.00)^a^
*Lp10*	24.00	16.00	53.47 (1.09)^a,b,c^	8358.40 (408.51)^a,b^	0.00 (0.00)^a^
*Lp11*	24.00	14.00	0.00 (0.00)a	21676.81 (7883.44)^a,b^	293.93 (55.08)^f^
*Lpl12*	24.00	16.00	48.47 (2.88)^a,b,c^	9584.65 (62.39)^a,b^	0.00 (0.00)^a^
*Lp13*	24.00	14.00	140.64 (27.46)^c^	81739.88 (19270.01)^d^	0.00 (0.00)^a^
*Lp14*	24.00	11.00	38.61 (11.53)^a,b^	6916.17 (1071.31)^a,b^	174.68 (30.01)^d,e^
*Lpl15*	24.00	15.00	0.00 (0.00)^a^	4434.58 (107.32)^a,b^	0.00 (0.00)^a^
*Lpl16*	24.00	13.00	12.36 (4.87)^a^	1122.13 (551.09)^a^	18.46 (7.73)^a,b^
*LcS*	22.00	14.00	ND	1197.50 (74.35)^a^	ND
*LrGG*	22.00	0.00	ND	11946.49 (3861.70)^a,b^	ND

*ND, not determined. Values are expressed as mean and standard deviation (in parentheses) obtained from triplicate experiments, except for the inhibition halo which test was not replicated. Different superscript letters, within the same column, means significant statistical differences (p ≤ 0.05) according to Sheffé post hoc comparison test. α-glucosidase, β-galactosidase, and phytase activities, as well as diameter of the inhibition halo (mm) produced on the food-borne pathogens E. coli ATTC 43894 (Ec) and L. monocytogenes NCTC 10357(Lm) test plate. Data were compared with the recognized probiotic microorganisms Lactobacillus casei Shirota (Lcs) and Lactobacillus rhamnosus GG (LrGG).*

Table 5 shows the susceptibility of the 16 LAB selected genotypes to many of the main antibiotics used in medicine. For the antibiotic concentrations assayed, many of the strains were very sensitive to the antibiotics, except for vancomycin, kanamycin, nalidixic acid, streptomycin, and cefotaxime, which were resistant. In general, they have similar behaviors than the probiotic microorganisms LcS and LrGG used as controls.

**Table 5:** Probiotic characteristics for the 16 predominant LAB genotypes obtained in the present study.

	Diameter of inhibition zone (mm)											
CEPAS/ANTB	E	T	CN	P	NA	AMP	S	VA	C	CD	K	FOX
*Lp1*	32S	21S	16I	27S	0R	37S	7R	0R	29S	39S	7R	7R
*Lp2*	32S	21S	17I	32S	0R	37S	6R	0R	29S	39S	7R	8R
*Lp3*	30S	20S	12R	28S	0R	25S	6R	0R	33S	40S	7R	8R
*Lp4*	29S	17I	14R	23S	0R	29S	6R	0R	26S	14R	7R	8R
*Lp5*	30S	20S	13R	16I	0R	33S	7R	0R	30S	7R	7R	6R
*Lp6*	27S	17I	9R	40S	0R	31S	6R	0R	29S	7R	0R	23S
*Lp7*	27S	20S	12R	20S	0R	30	7R	0R	26S	12R	6R	6R
*Lp8*	27S	20S	14R	20S	0R	31S	7R	0R	27S	8R	7R	6R
*LPG1*	27S	19I	15I	22S	0R	31S	6R	0R	26S	9R	7R	6R
*Lp10*	26S	19I	13R	16I	0R	30S	6R	0R	28S	11R	6R	7R
*Lp11*	29S	21S	16I	25S	0R	24S	6R	0R	28S	8R	7R	7R
*Lpl12*	30S	20S	13R	23S	0R	35S	0R	0R	25S	13R	0R	8R
*Lp13*	27S	18I	15I	23S	0R	32S	6R	0R	29S	8R	6R	7R
*Lp14*	31S	22S	19I	28S	0R	32S	12R	0R	29S	35S	8R	8R
*Lpl15*	30S	20S	11R	22S	0R	32S	0R	0R	28S	8R	0R	11R
*Lpl16*	30S	17I	13R	26S	0R	35S	6R	0R	31S	8R	7R	16I
*LcS*	28S	20S	11R	22S	0R	35S	7R	0R	29S	33S	0R	8R
*LrGG*	27S	20S	11R	21S	0R	37S	7R	0R	29S	31S	0R	7R

*Values are expressed as diameter of the inhibition zone (mm) for the antibiotics E, erythromycin (15 μg); TE, tetracycline; CN, gentamicin (10 μg); P, penicillin (10 μg); NA, nalidixic acid (30 μg); AMP, ampicillin (10 μg); S, streptomycin (10 μg); VA, vancomycin (30 μg); C, chloramphenicol (30 μg); CD, clindamycin (2 μg); K, kanamycin (30 μg) and FOX, cefotaxime (30 μg). The letters indicate: S, susceptible (zone diameter ≥ 20 mm); I, intermediate (zone diameter, 15–19 mm); and R, resistant (zone diameter ≤ 14), according to the standard given by Clinical and Laboratory Standards Institute (2012). Antibiotic susceptibility, determined by diffusion assay, against the high consumption antibiotics.*

### Multivariate Analysis

**FIGURE 3:**
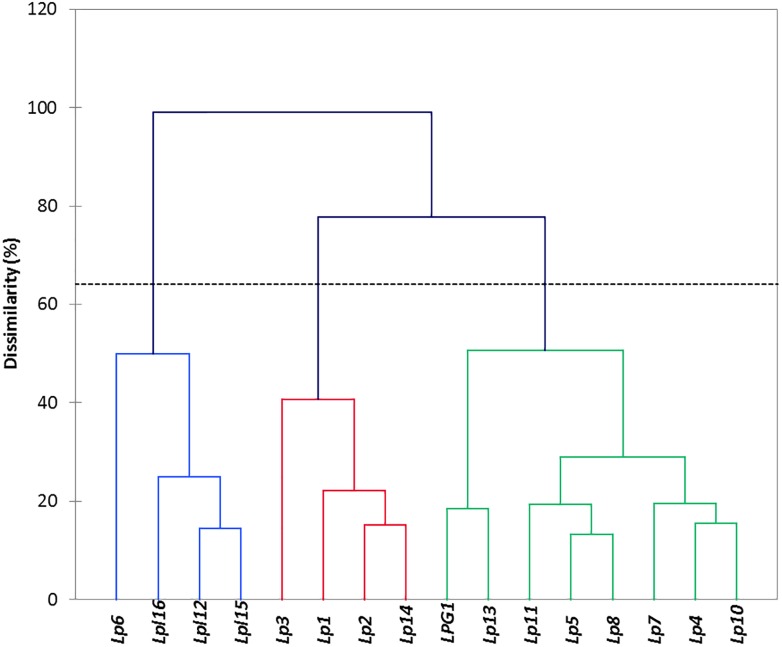
Clustering obtained after multivariate analysis using the XLSTAT software. Cluster indicates the relation between genotypes, based on the results of the probiotics and technological tests.

The automatic selection of clusters base on genotype entropy (Figure 3), revealed the presence of three main groups, which profiles differed mainly (most discriminatory variables) on the production of lactic acid, α-glucosidase, the presence of *bsh1* gene, gastric and pancreatic digestion. This way, there were two groups composed of four elements; the first one (Lp6, Lpl16, LPL12, and Lpl15) included all the *L. plantarum* genotypes, which showed very similar behaviours among them but had a great dissimilarity with the rest of *L. pentosus* genotypes (except Lp6). The second cluster included Lp1, Lp2, Lp3, and Lp14 genotypes with low contrast among them and, apparently, with neither of them representing all the group properties. Finally, the third group included the rest of *L. pentosus* genotypes, with high similarities among some of them but finding different sub-clusters (LPG1 and Lp13; Lp11, Lp5, and Lp8; Lp7, Lp4, and Lp10). Therefore, the clusters could be a useful tool for a further selection of the genotypes used for new expected starter cultures.

**FIGURE 4:**
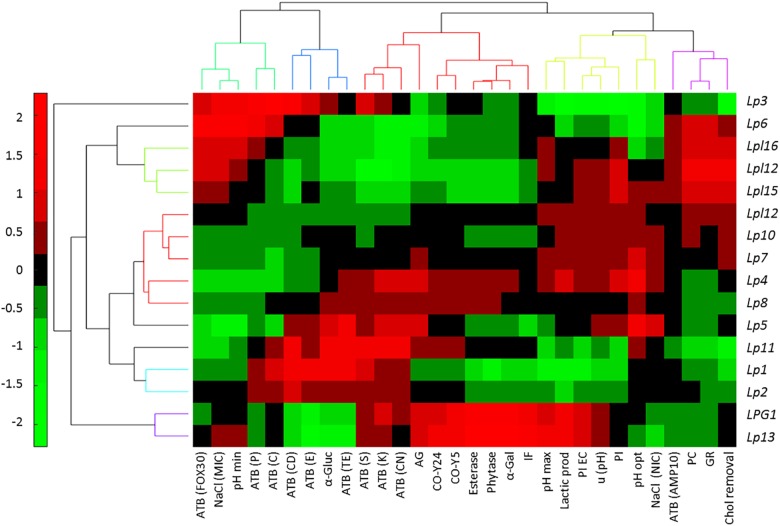
Bicluster obtained after a multivariate analysis of genotypes, based on the probiotics and technological tests, using the Multibiplot Software. Cells’ colors represent the genotype contribution to each variable. The meanings of the abbreviations are: ATB(FOX30), antibiotic cefotaxime; NACl(MIC), minimum inhibitory concentration of sodium chloride; pH (min), minimum pH value; ATB(P10), penicillin antibiotic; ATB(C30), chloramphenicol antibiotic; ATB(CD2), clindamycin antibiotic; ATB(E15), erythromycin antibiotic; α-Gluc, α-glucosidase; ATB(TE), tetracycline antibiotic; ATB(S10); streptomycin antibiotic; ATB(K30), kanamycin antibiotic; ATB(CN10), gentamycin antibiotic; AG, auto-aggregation; CO-Y24, co-aggregation with yeast Y24; CO-Y5, co-aggregation with yeast Y5; Esterase; Phytase; α-Galact, α-galactosidase; IF, isolation frequency; pH_max_, maximum growth pH; Lactic prod, Lactic acid production; PI EC; *E. coli* inhibition; u (pH), maximum area obtained for pH; PL, *Listeria monocytogenes* inhibition; pH_opt_, optimum growth pH; NaCl(NIC), non-inhibitory concentration of sodium chloride; ATB(AMP10), ampicillin antibiotic; PC, pancreatic digestion; GR, gastric digestion; Chol removal, cholesterol removal.

The bicluster analysis has the advantage over the unidimensional cluster of relating each genotype with their characteristics allowing the assessment of their properties in a glance. In this analysis, the first appreciation is the clear segregation of Lp3 over the rest of genotypes because of its high susceptibility respecting the other strains to the antibiotics clindamycin, erythromycin, and streptomycin, and low values of resistance to pancreatic, gastric digestion, and cholesterol removal (Figure 4). However, the second cluster (Lp6, Lpl16, Lpl12, and Lpl15) is similar to that previously observed in Figure 3 and is characterized by the higher susceptibility to the antibiotic cefotaxime and resistance to antibiotics tetracycline, streptomycin, kanamycin, and gentamycin, resistance to NaCl, pancreatic and gastric digestion, and cholesterol removal, while show rather low values of α-glucosidase, auto-aggregation and co-aggregation with yeasts, esterase, phytase, and α-galactosidase activities. Also, the cluster composed of LPG1 and Lp13 is very homogeneous and presents high values for auto and co-aggregation with yeasts, esterase, phytase, and α-galactosidase activities, isolation frequency, pH_max_, lactic acid production, and inhibition of *E. coli*. The other big cluster is not as homogeneous as the previous ones commented. Although Lp10 and Lp7 had moderate high values in pH_max_, lactic acid production, inhibition of *E. coli* and *L. monocytogenes*, u(pH), and pH_opt_; conversely, most of the rest of genotypes included in this cluster had moderate to high values of α-glucosidase and auto-aggregation and susceptibility to the antibiotics penicillin, chloramphenicol, clindamycin, erythromycin, streptomycin, kanamycin, and gentamicin. Hence, Figure 4 presents a picture (heat map) able to guide the selection of the genotype according to the properties one expects from the starter culture.

Regarding technological or probiotic characteristics, four clusters are observed. The first one, from left to right (Figure 4, upper) includes the antibiotics cefotaxime, penicillin and chloramphenicol, NaCl (MIC), and pH_min_; the second, antibiotics clindamycin, erythromycin, and tetracycline, as well as α-glucosidase activity; the third and fourth includes successively following the 9 and 11 characteristics, respectively. Clustering some variables may indicate the simultaneous presence in specific genotypes. Looking for their relationships is outside the scope of this work, but such association opens a possible line of research expected to be explored in the future.

## Discussion

A step-by step procedure which comprises biofilm detachment, isolation, genotyping, identification, screening of technological and probiotic features, and use of multivariate analysis, was used in the present work for the study of the LAB biodiversity present in table olive biofilms and selection of the most promising strains for their use as starter cultures. Most of the recent works focusing on the study of the bacterial biodiversity in table olives have been carried out isolating microorganisms from olives brines, finding that *L. pentosus* and *L. plantarum* were the dominant species among LAB (Abriouel et al., 2012; Lucena-Padrós et al., 2014a,b; Tofalo et al., 2014; Comunian et al., 2017). However, scarce studies have been carried out to exclusively study the LAB biodiversity in the olive epidermis albeit these microorganisms would be directly transferred to human during olive consumption. The study of microorganisms associated to olive epidermis is more complex than in brines, because detachment of cells from mature biofilms may be not complete and therefore microbial counts would be underestimated. Benítez-Cabello et al. (2016) reported by independent culture methods (RT-PCR-DGGE) the presence of the *L. plantarum* group, *Lactobacillus sanfranciscensis*, and *Lactobacillus parafarraginis* in biofilms of Manzanilla and Gordal olives processed as Spanish-style. The same research group found that *L. pentosus* was the dominant species not only in the biofilms of directly brined Gordal olives (Benítez-Cabello et al., 2015) but also in the same cultivar when processed as Spanish-style (Domínguez-Manzano et al., 2012). On the contrary, Cocolin et al. (2013) reported that *L. plantarum* was the dominant LAB species present in the biofilms of Italian olives (Nocellare etnea cultivar) processed both as lye-treated or natural olives. The number of genotypes (biodiversity) for *L. plantarum* was higher in natural olives than in lye-treated olives (Cocolin et al., 2013). This results contrast with the data obtained in the present study, where the presence of LAB genotypes was higher in the Spanish-style than in natural olives. In directly brined processes, hydrolysis of phenolic compounds is achieved more slowly than in lye-treated olives (Garrido-Fernández et al., 1997). Many of these degraded phenolic compounds are powerful antibacterial compounds which hinder the growth of LAB species during olive fermentation (Medina et al., 2010). Therefore, the growth of LAB species (at least for *L. pentosus*) in not lye-treated olives may be limited to only the LAB genotypes with higher resistance to phenolic compounds, characteristic that reduces their biodiversity. The behavior would also be modulated by the concentrations of such compounds in olives and brines which will depend on the type of table olive style, elaboration and also differs among olive cultivars (Medina et al., 2010).

Lucena-Padrós et al. (2014a) performed an interesting study of the microbial genetic diversity in Spanish-style fermentations by RAPD-PCR. Among a total of 638 LAB isolates obtained from brines in two industries, they found 144 different genotypes, the most assigned to *L. pentosus* species, but few of them also belong to *L. plantarum* and *L. paraplantarum* species. This contrast with data obtained in the present study. A total of 79 genotypes were discriminated among 554 LAB isolates obtained from biofilms of different industries, cultivars and styles. This work demonstrates that the selective environment governing table olive biofilms may induce lower genetic biodiversity than brines, due to the circumstance that not all LAB genotypes can adhere to fruit epidermis.

The 16 predominant LAB genotypes obtained from olives biofilms were then subjected to technological and probiotic tests for selection of the best multifunctional starters. Previous studies have proved the existence in table olive fermentations of LAB strains with potential probiotic characteristics, isolated from Spanish (Bautista-Gallego et al., 2013), Italian (Botta et al., 2014), Portuguese (Peres et al., 2014), or Greek (Argyri et al., 2013) olive brine fermentations. However, no study has been carried out till now specifically with strains isolated from table olive biofilms. This is a very exciting issue because the adherence is an important requirement for selection of LAB strains with probiotic potential since one should prove the ability of the selected strain to adhere to the olive surface, turning olives as a delivery vehicle of probiotic microorganisms to consumers (Arroyo-López et al., 2015).

Multivariate analysis was used for grouping genotypes as a function of their probiotic and technological features. This statistical approach is appropriate when researchers must manage and analyze a large amount of data of a considerable number of genotypes. In the last years, many researchers have used this methodology for the selection of the most promising starters for table olive processing, using principal component analysis or hierarchical cluster analysis to reach their goals (Bautista-Gallego et al., 2013; Bevilacqua et al., 2013; Botta et al., 2014; Porru et al., 2018). In this work, not only the genotypes were clustered based on their overall characteristics, but also the bicluster mapped the properties that each group had as well as the individual characterisation of the diverse strains. The initial 16 genotypes were reduced to only 3 great phenotypes, one that included the *L. plantarum* genotypes group (plus Lp6 strain) and other 2 clusters with the rest of *L. pentosus* genotypes, according with the low biodiversity observed. However, the bicluster has shown that even considering the low general variability; the selected genotypes have individual specific characteristics which may be of particular interest for special cases (e.g., *L. plantarum* for lowering the cholesterol levels or *L. pentosus* Lp13 and LpG1 for their high levels of esterase and phytase activities).

Therefore, the selection of a single strain which had the best or highest values for all characteristics is a great challenge. If one would have to choose among the selected LAB genotypes only one for its use as multifunctional starter, maybe *L. pentosus* Lp13 (or LPG1) would be the most attractive because, in addition to a good performance for many of the technological and probiotic tests, was the genotype more frequently found (presumably because of its high imposition frequency during fermentation) as well as its high titratable acidity production (homofermentative metabolism), possibility of inoculation at high pH levels, esterase (production of aromas and degradation of bitter compounds), β-galactosidase (important in lactose assimilation), and phytase activity (necessary for assimilation of phosphate and other minerals).

## Conclusion

*Lactobacillus pentosus* was the predominant species found at industrial scale in Spanish table olives biofilms, albeit certain genotypes of *L. plantarum* were also detected. At genotype level, biodiversity was higher in the Spanish-style table olive biofilms than in those of directly brined olives. The multivariate analysis based on technological and probiotic data showed that the main 16 genotypes obtained could be clustered in 3 great phenotypes, with some strains with potential application as multifunctional starters (especially *L. pentosus* Lp13 and LpG1 genotypes). Data obtained in the present study showed the selective environment that governs table olive biofilms, where not all LAB genotypes has the ability to adhere on and, as a result, the reduced biodiversity of its flora.

## Data Availability

No datasets were generated or analyzed for this study.

## Author Contributions

AB-C carried out the experimental work and helped in the writing of the manuscript. BC-D assisted in part of the experimental work. FR-G led part of the general design of the experiments and assisted in the experimental work. RJ-D contributed in the general design of the experiments and supervision. AG-F carried out the statistical analysis and also helped in the writing of the manuscript. FA-L supervised and contributed in the general design of the experiments and wrote the manuscript.

## Conflict of Interest Statement

The authors declare that the research was conducted in the absence of any commercial or financial relationships that could be construed as a potential conflict of interest.

## Footnotes

1 http://blast.ncbi.nlm.nih.gov/Blast.cgi
